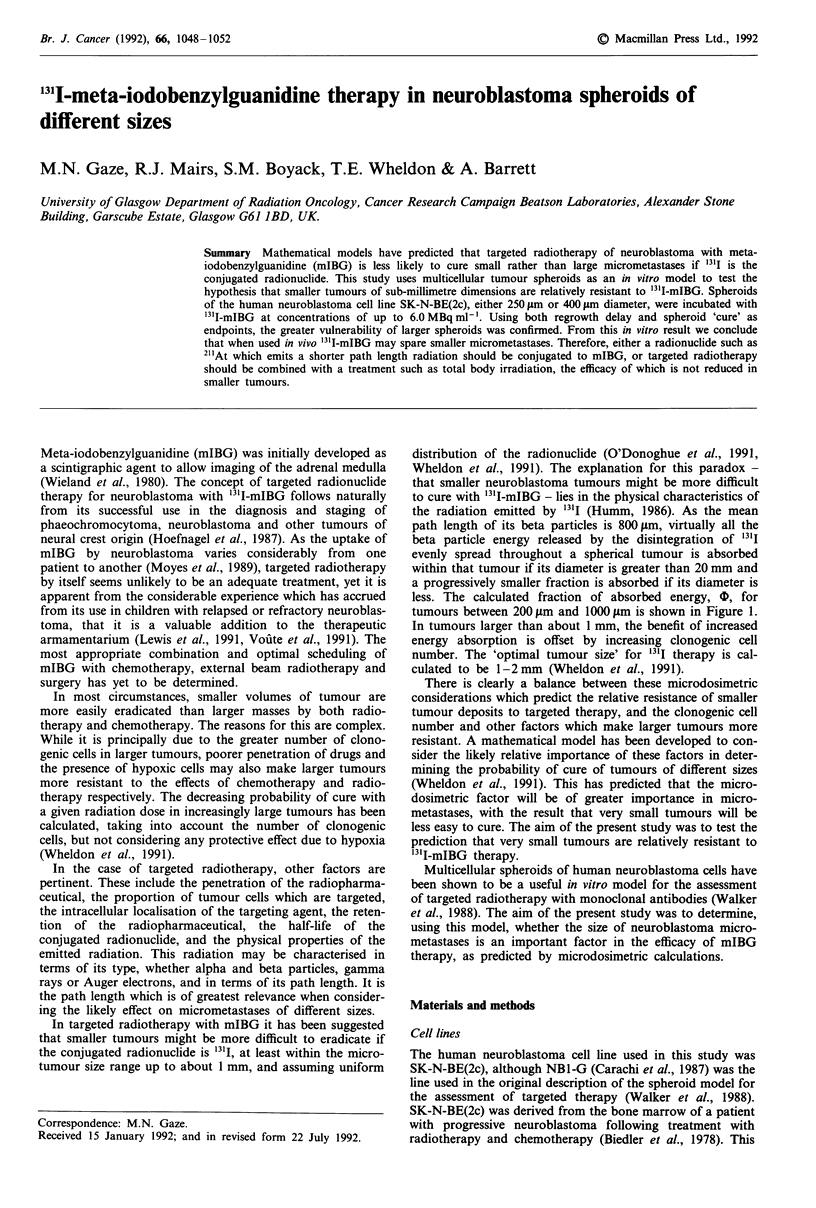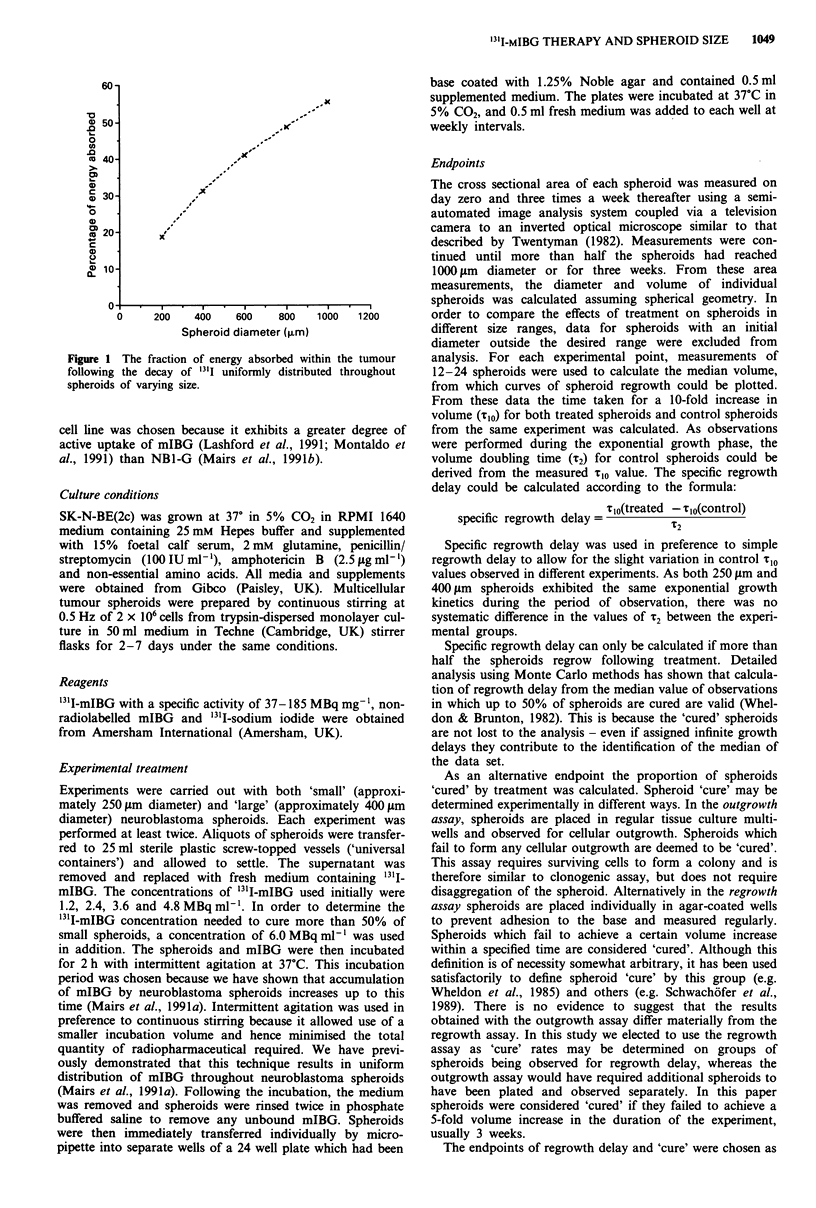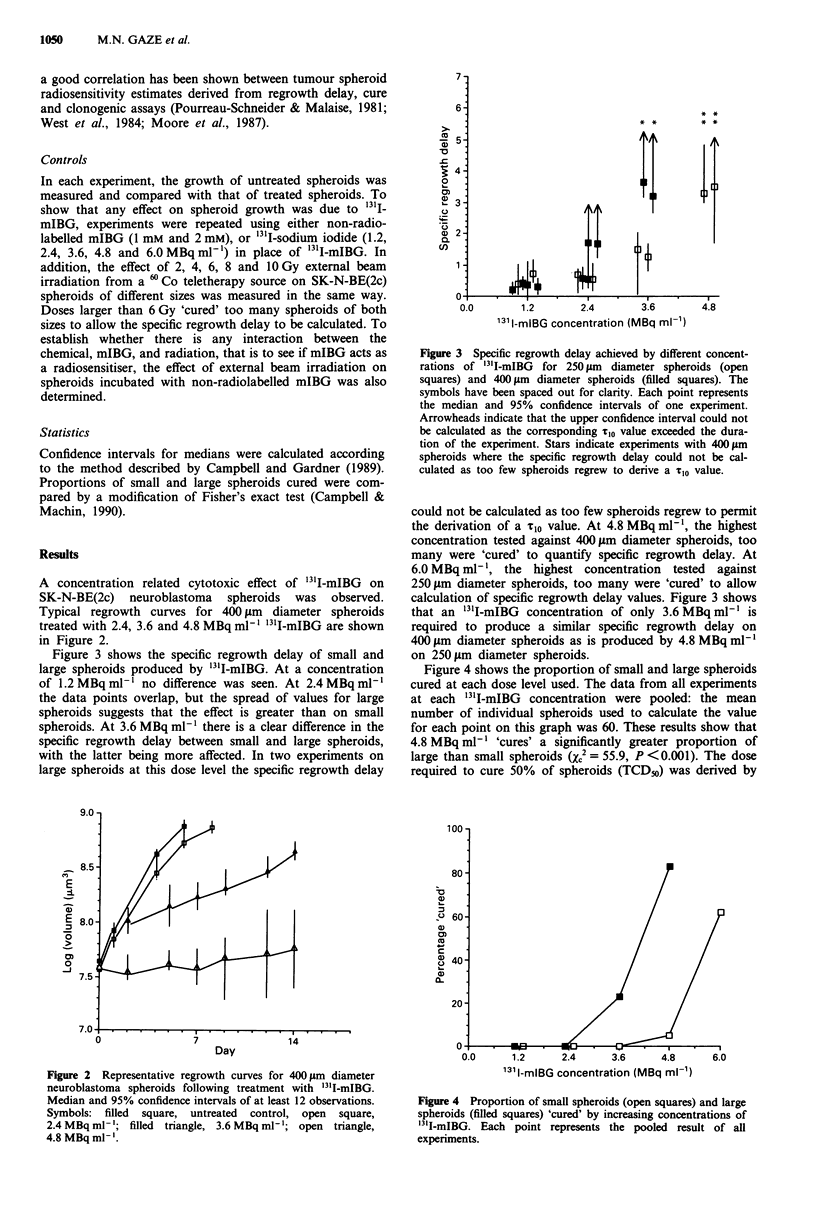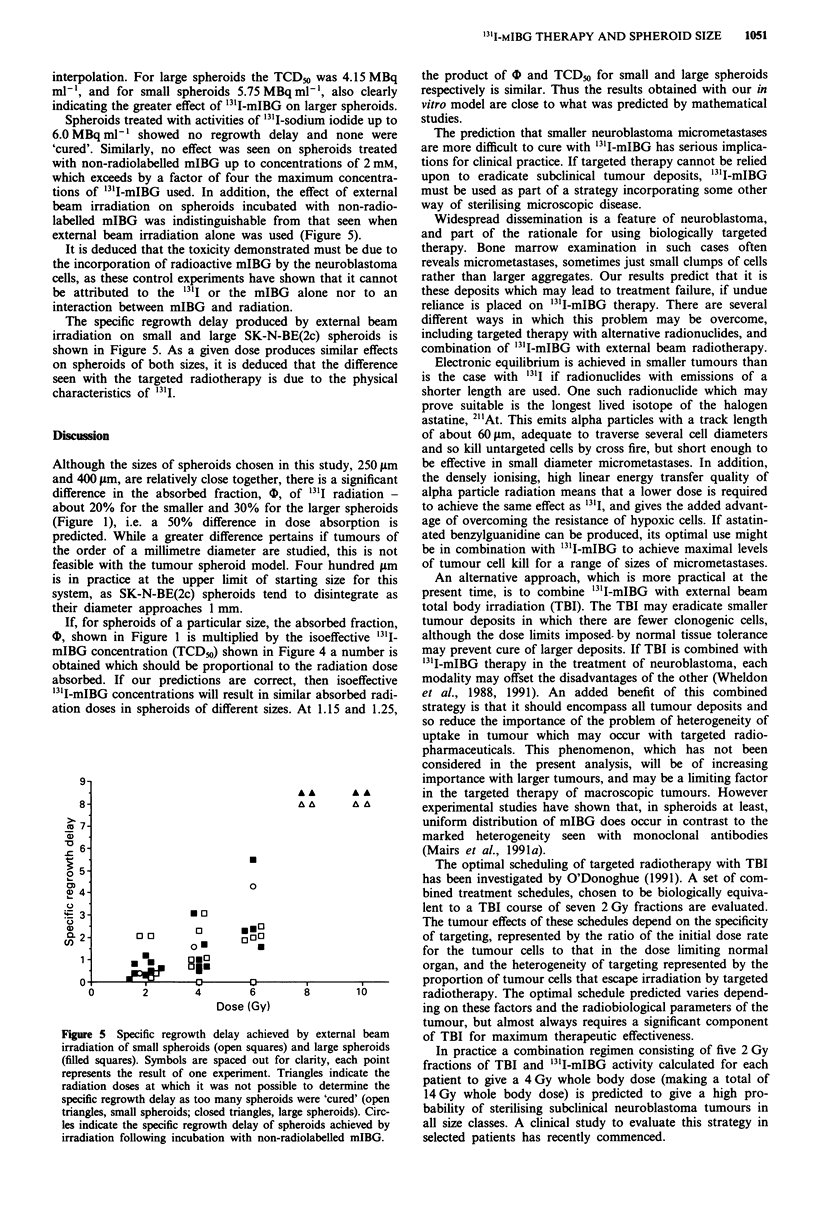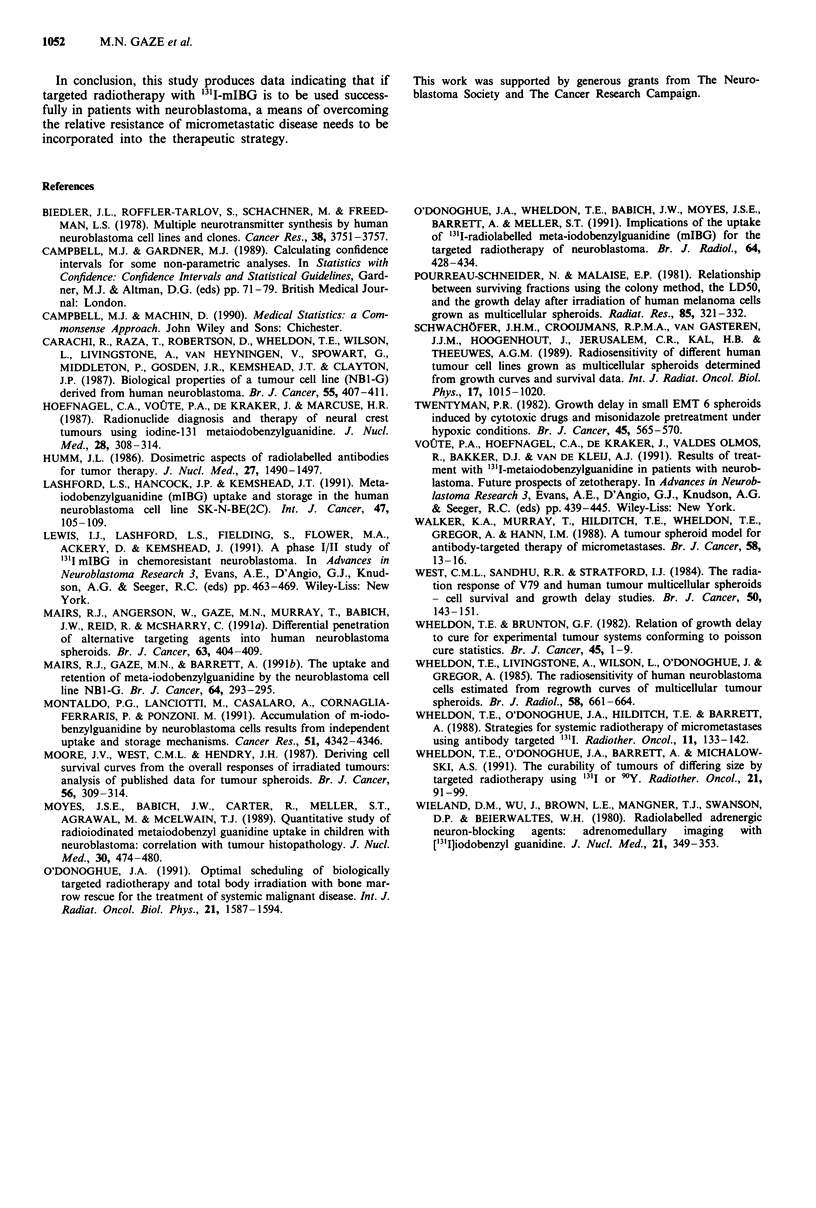# 131I-meta-iodobenzylguanidine therapy in neuroblastoma spheroids of different sizes.

**DOI:** 10.1038/bjc.1992.408

**Published:** 1992-12

**Authors:** M. N. Gaze, R. J. Mairs, S. M. Boyack, T. E. Wheldon, A. Barrett

**Affiliations:** University of Glasgow Department of Radiation Oncology, Cancer Research Campaign Beatson Laboratories, Glasgow, UK.

## Abstract

Mathematical models have predicted that targeted radiotherapy of neuroblastoma with metaiodobenzylguanidine (mIBG) is less likely to cure small rather than large micrometastases if 131I is the conjugated radionuclide. This study uses multicellular tumour spheroids as an in vitro model to test the hypothesis that smaller tumours of sub-millimetre dimensions are relatively resistant to 131I-mIBG. Spheroids of the human neuroblastoma cell line SK-N-BE(2c), either 250 microns or 400 microns diameter, were incubated with 131I-mIBG at concentrations of up to 6.0 MBq ml-1. Using both regrowth delay and spheroid 'cure' as endpoints, the greater vulnerability of larger spheroids was confirmed. From this in vitro result we conclude that when used in vivo 131I-mIBG may spare smaller micrometastases. Therefore, either a radionuclide such as 211At which emits a shorter path length radiation should be conjugated to mIBG, or targeted radiotherapy should be combined with a treatment such as total body irradiation, the efficacy of which is not reduced in smaller tumours.


Br. J. Cancer (1992), 66, 1048–1052 © Macmillan Press Ltd., 1992

### $^{131}$I-meta-iodobenzylguanidine therapy in neuroblastoma spheroids of different sizes

M.N. Gaze, R.J. Mairs, S.M. Boyack, T.E. Wheldon & A. Barrett

*University of Glasgow Department of Radiation Oncology, Cancer Research Campaign Beatson Laboratories, Alexander Stone Building, Garscube Estate, Glasgow G61 1BD, UK.*

**Summary** Mathematical models have predicted that targeted radiotherapy of neuroblastoma with meta-iodobenzylguanidine (mIBG) is less likely to cure small rather than large micrometastases if $^{131}$I is the conjugated radionuclide. This study uses multicellular tumour spheroids as an *in vitro* model to test the hypothesis that smaller tumours of sub-millimetre dimensions are relatively resistant to $^{131}$I-mIBG. Spheroids of the human neuroblastoma cell line SK-N-BE(2c), either 250 µm or 400 µm diameter, were incubated with $^{131}$I-mIBG at concentrations of up to 6.0 MBq ml$^{-1}$. Using both regrowth delay and spheroid 'cure' as endpoints, the greater vulnerability of larger spheroids was confirmed. From this *in vitro* result we conclude that when used *in vivo* $^{131}$I-mIBG may spare smaller micrometastases. Therefore, either a radionuclide such as $^{211}$At which emits a shorter path length radiation should be conjugated to mIBG, or targeted radiotherapy should be combined with a treatment such as total body irradiation, the efficacy of which is not reduced in smaller tumours.

Meta-iodobenzylguanidine (mIBG) was initially developed as a scintigraphic agent to allow imaging of the adrenal medulla (Wieland *et al.*, 1980). The concept of targeted radionuclide therapy for neuroblastoma with $^{131}$I-mIBG follows naturally from its successful use in the diagnosis and staging of phaeochromocytoma, neuroblastoma and other tumours of neural crest origin (Hoefnagel *et al.*, 1987). As the uptake of mIBG by neuroblastoma varies considerably from one patient to another (Moyes *et al.*, 1989), targeted radiotherapy by itself seems unlikely to be an adequate treatment, yet it is apparent from the considerable experience which has accrued from its use in children with relapsed or refractory neuroblastoma, that it is a valuable addition to the therapeutic armamentarium (Lewis *et al.*, 1991, Voûte *et al.*, 1991). The most appropriate combination and optimal scheduling of mIBG with chemotherapy, external beam radiotherapy and surgery has yet to be determined.

In most circumstances, smaller volumes of tumour are more easily eradicated than larger masses by both radiotherapy and chemotherapy. The reasons for this are complex. While it is principally due to the greater number of clonogenic cells in larger tumours, poorer penetration of drugs and the presence of hypoxic cells may also make larger tumours more resistant to the effects of chemotherapy and radiotherapy respectively. The decreasing probability of cure with a given radiation dose in increasingly large tumours has been calculated, taking into account the number of clonogenic cells, but not considering any protective effect due to hypoxia (Wheldon *et al.*, 1991).

In the case of targeted radiotherapy, other factors are pertinent. These include the penetration of the radiopharmaceutical, the proportion of tumour cells which are targeted, the intracellular localisation of the targeting agent, the retention of the radiopharmaceutical, the half-life of the conjugated radionuclide, and the physical properties of the emitted radiation. This radiation may be characterised in terms of its type, whether alpha and beta particles, gamma rays or Auger electrons, and in terms of its path length. It is the path length which is of greatest relevance when considering the likely effect on micrometastases of different sizes.

In targeted radiotherapy with mIBG it has been suggested that smaller tumours might be more difficult to eradicate if the conjugated radionuclide is $^{131}$I, at least within the micro-tumour size range up to about 1 mm, and assuming uniform distribution of the radionuclide (O'Donoghue *et al.*, 1991, Wheldon *et al.*, 1991). The explanation for this paradox – that smaller neuroblastoma tumours might be more difficult to cure with $^{131}$I-mIBG – lies in the physical characteristics of the radiation emitted by $^{131}$I (Humm, 1986). As the mean path length of its beta particles is 800 µm, virtually all the beta particle energy released by the disintegration of $^{131}$I evenly spread throughout a spherical tumour is absorbed within that tumour if its diameter is greater than 20 mm and a progressively smaller fraction is absorbed if its diameter is less. The calculated fraction of absorbed energy, $\Phi$, for tumours between 200 µm and 1000 µm is shown in Figure 1. In tumours larger than about 1 mm, the benefit of increased energy absorption is offset by increasing clonogenic cell number. The 'optimal tumour size' for $^{131}$I therapy is calculated to be 1–2 mm (Wheldon *et al.*, 1991).

There is clearly a balance between these microdosimetric considerations which predict the relative resistance of smaller tumour deposits to targeted therapy, and the clonogenic cell number and other factors which make larger tumours more resistant. A mathematical model has been developed to consider the likely relative importance of these factors in determining the probability of cure of tumours of different sizes (Wheldon *et al.*, 1991). This has predicted that the microdosimetric factor will be of greater importance in micrometastases, with the result that very small tumours will be less easy to cure. The aim of the present study was to test the prediction that very small tumours are relatively resistant to $^{131}$I-mIBG therapy.

Multicellular spheroids of human neuroblastoma cells have been shown to be a useful *in vitro* model for the assessment of targeted radiotherapy with monoclonal antibodies (Walker *et al.*, 1988). The aim of the present study was to determine, using this model, whether the size of neuroblastoma micrometastases is an important factor in the efficacy of mIBG therapy, as predicted by microdosimetric calculations.

### Materials and methods

### *Cell lines*

The human neuroblastoma cell line used in this study was SK-N-BE(2c), although NB1-G (Carachi *et al.*, 1987) was the line used in the original description of the spheroid model for the assessment of targeted therapy (Walker *et al.*, 1988). SK-N-BE(2c) was derived from the bone marrow of a patient with progressive neuroblastoma following treatment with radiotherapy and chemotherapy (Biedler *et al.*, 1978). This

Correspondence: M.N. Gaze.
Received 15 January 1992; and in revised form 22 July 1992.

**Figure 1** The fraction of energy absorbed within the tumour following the decay of $^{131}$I uniformly distributed throughout spheroids of varying size.

cell line was chosen because it exhibits a greater degree of active uptake of mIBG (Lashford *et al.*, 1991; Montaldo *et al.*, 1991) than NB1-G (Mairs *et al.*, 1991*b*).

### *Culture conditions*

SK-N-BE(2c) was grown at 37° in 5% $CO_2$ in RPMI 1640 medium containing 25 mM Hepes buffer and supplemented with 15% foetal calf serum, 2 mM glutamine, penicillin/streptomycin (100 IU $ml^{-1}$), amphotericin B (2.5 μg $ml^{-1}$) and non-essential amino acids. All media and supplements were obtained from Gibco (Paisley, UK). Multicellular tumour spheroids were prepared by continuous stirring at 0.5 Hz of $2 \times 10^6$ cells from trypsin-dispersed monolayer culture in 50 ml medium in Techne (Cambridge, UK) stirrer flasks for 2–7 days under the same conditions.

### *Reagents*

$^{131}$I-mIBG with a specific activity of 37–185 MBq $mg^{-1}$, non-radiolabelled mIBG and $^{131}$I-sodium iodide were obtained from Amersham International (Amersham, UK).

### *Experimental treatment*

Experiments were carried out with both 'small' (approximately 250 μm diameter) and 'large' (approximately 400 μm diameter) neuroblastoma spheroids. Each experiment was performed at least twice. Aliquots of spheroids were transferred to 25 ml sterile plastic screw-topped vessels ('universal containers') and allowed to settle. The supernatant was removed and replaced with fresh medium containing $^{131}$I-mIBG. The concentrations of $^{131}$I-mIBG used initially were 1.2, 2.4, 3.6 and 4.8 MBq $ml^{-1}$. In order to determine the $^{131}$I-mIBG concentration needed to cure more than 50% of small spheroids, a concentration of 6.0 MBq $ml^{-1}$ was used in addition. The spheroids and mIBG were then incubated for 2 h with intermittent agitation at 37°C. This incubation period was chosen because we have shown that accumulation of mIBG by neuroblastoma spheroids increases up to this time (Mairs *et al.*, 1991*a*). Intermittent agitation was used in preference to continuous stirring because it allowed use of a smaller incubation volume and hence minimised the total quantity of radiopharmaceutical required. We have previously demonstrated that this technique results in uniform distribution of mIBG throughout neuroblastoma spheroids (Mairs *et al.*, 1991*a*). Following the incubation, the medium was removed and spheroids were rinsed twice in phosphate buffered saline to remove any unbound mIBG. Spheroids were then immediately transferred individually by micropipette into separate wells of a 24 well plate which had been base coated with 1.25% Noble agar and contained 0.5 ml supplemented medium. The plates were incubated at 37°C in 5% $CO_2$, and 0.5 ml fresh medium was added to each well at weekly intervals.

### *Endpoints*

The cross sectional area of each spheroid was measured on day zero and three times a week thereafter using a semi-automated image analysis system coupled via a television camera to an inverted optical microscope similar to that described by Twentyman (1982). Measurements were continued until more than half the spheroids had reached 1000 μm diameter or for three weeks. From these area measurements, the diameter and volume of individual spheroids was calculated assuming spherical geometry. In order to compare the effects of treatment on spheroids in different size ranges, data for spheroids with an initial diameter outside the desired range were excluded from analysis. For each experimental point, measurements of 12–24 spheroids were used to calculate the median volume, from which curves of spheroid regrowth could be plotted. From these data the time taken for a 10-fold increase in volume ($\tau_{10}$) for both treated spheroids and control spheroids from the same experiment was calculated. As observations were performed during the exponential growth phase, the volume doubling time ($\tau_2$) for control spheroids could be derived from the measured $\tau_{10}$ value. The specific regrowth delay could be calculated according to the formula:

$$\text{specific regrowth delay} = \frac{\tau_{10}(\text{treated} \ - \tau_{10}(\text{control})}{\tau_2}$$

Specific regrowth delay was used in preference to simple regrowth delay to allow for the slight variation in control $\tau_{10}$ values observed in different experiments. As both 250 μm and 400 μm spheroids exhibited the same exponential growth kinetics during the period of observation, there was no systematic difference in the values of $\tau_2$ between the experimental groups.

Specific regrowth delay can only be calculated if more than half the spheroids regrow following treatment. Detailed analysis using Monte Carlo methods has shown that calculation of regrowth delay from the median value of observations in which up to 50% of spheroids are cured are valid (Wheldon & Brunton, 1982). This is because the 'cured' spheroids are not lost to the analysis – even if assigned infinite growth delays they contribute to the identification of the median of the data set.

As an alternative endpoint the proportion of spheroids 'cured' by treatment was calculated. Spheroid 'cure' may be determined experimentally in different ways. In the *outgrowth assay*, spheroids are placed in regular tissue culture multiwells and observed for cellular outgrowth. Spheroids which fail to form any cellular outgrowth are deemed to be 'cured'. This assay requires surviving cells to form a colony and is therefore similar to clonogenic assay, but does not require disaggregation of the spheroid. Alternatively in the *regrowth assay* spheroids are placed individually in agar-coated wells to prevent adhesion to the base and measured regularly. Spheroids which fail to achieve a certain volume increase within a specified time are considered 'cured'. Although this definition is of necessity somewhat arbitrary, it has been used satisfactorily to define spheroid 'cure' by this group (e.g. Wheldon *et al.*, 1985) and others (e.g. Schwachöfer *et al.*, 1989). There is no evidence to suggest that the results obtained with the outgrowth assay differ materially from the regrowth assay. In this study we elected to use the regrowth assay as 'cure' rates may be determined on groups of spheroids being observed for regrowth delay, whereas the outgrowth assay would have required additional spheroids to have been plated and observed separately. In this paper spheroids were considered 'cured' if they failed to achieve a 5-fold volume increase in the duration of the experiment, usually 3 weeks.

The endpoints of regrowth delay and 'cure' were chosen as a good correlation has been shown between tumour spheroid radiosensitivity estimates derived from regrowth delay, cure and clonogenic assays (Pourreau-Schneider & Malaise, 1981; West et al., 1984; Moore et al., 1987).

*Controls*

In each experiment, the growth of untreated spheroids was measured and compared with that of treated spheroids. To show that any effect on spheroid growth was due to $^{131}$I-mIBG, experiments were repeated using either non-radiolabelled mIBG (1 mM and 2 mM), or $^{131}$I-sodium iodide (1.2, 2.4, 3.6, 4.8 and 6.0 MBq ml$^{-1}$) in place of $^{131}$I-mIBG. In addition, the effect of 2, 4, 6, 8 and 10 Gy external beam irradiation from a $^{60}$Co teletherapy source on SK-N-BE(2c) spheroids of different sizes was measured in the same way. Doses larger than 6 Gy 'cured' too many spheroids of both sizes to allow the specific regrowth delay to be calculated. To establish whether there is any interaction between the chemical, mIBG, and radiation, that is to see if mIBG acts as a radiosensitiser, the effect of external beam irradiation on spheroids incubated with non-radiolabelled mIBG was also determined.

*Statistics*

Confidence intervals for medians were calculated according to the method described by Campbell and Gardner (1989). Proportions of small and large spheroids cured were compared by a modification of Fisher's exact test (Campbell & Machin, 1990).

### Results

A concentration related cytotoxic effect of $^{131}$I-mIBG on SK-N-BE(2c) neuroblastoma spheroids was observed. Typical regrowth curves for 400 μm diameter spheroids treated with 2.4, 3.6 and 4.8 MBq ml$^{-1}$ $^{131}$I-mIBG are shown in Figure 2.

Figure 3 shows the specific regrowth delay of small and large spheroids produced by $^{131}$I-mIBG. At a concentration of 1.2 MBq ml$^{-1}$ no difference was seen. At 2.4 MBq ml$^{-1}$ the data points overlap, but the spread of values for large spheroids suggests that the effect is greater than on small spheroids. At 3.6 MBq ml$^{-1}$ there is a clear difference in the specific regrowth delay between small and large spheroids, with the latter being more affected. In two experiments on large spheroids at this dose level the specific regrowth delay could not be calculated as too few spheroids regrew to permit the derivation of a $\tau_{10}$ value. At 4.8 MBq ml$^{-1}$, the highest concentration tested against 400 μm diameter spheroids, too many were 'cured' to quantify specific regrowth delay. At 6.0 MBq ml$^{-1}$, the highest concentration tested against 250 μm diameter spheroids, too many were 'cured' to allow calculation of specific regrowth delay values. Figure 3 shows that an $^{131}$I-mIBG concentration of only 3.6 MBq ml$^{-1}$ is required to produce a similar specific regrowth delay on 400 μm diameter spheroids as is produced by 4.8 MBq ml$^{-1}$ on 250 μm diameter spheroids.

Figure 4 shows the proportion of small and large spheroids cured at each dose level used. The data from all experiments at each $^{131}$I-mIBG concentration were pooled: the mean number of individual spheroids used to calculate the value for each point on this graph was 60. These results show that 4.8 MBq ml$^{-1}$ 'cures' a significantly greater proportion of large than small spheroids ($\chi_c^2 = 55.9$, $P < 0.001$). The dose required to cure 50% of spheroids (TCD$_{50}$) was derived by

**Figure 3** Specific regrowth delay achieved by different concentrations of $^{131}$I-mIBG for 250 μm diameter spheroids (open squares) and 400 μm diameter spheroids (filled squares). The symbols have been spaced out for clarity. Each point represents the median and 95% confidence intervals of one experiment. Arrowheads indicate that the upper confidence interval could not be calculated as the corresponding $\tau_{10}$ value exceeded the duration of the experiment. Stars indicate experiments with 400 μm spheroids where the specific regrowth delay could not be calculated as too few spheroids regrew to derive a $\tau_{10}$ value.

**Figure 2** Representative regrowth curves for 400 μm diameter neuroblastoma spheroids following treatment with $^{131}$I-mIBG. Median and 95% confidence intervals of at least 12 observations. Symbols: filled square, untreated control, open square, 2.4 MBq ml$^{-1}$; filled triangle, 3.6 MBq ml$^{-1}$; open triangle, 4.8 MBq ml$^{-1}$.

**Figure 4** Proportion of small spheroids (open squares) and large spheroids (filled squares) 'cured' by increasing concentrations of $^{131}$I-mIBG. Each point represents the pooled result of all experiments.

interpolation. For large spheroids the $TCD_{50}$ was 4.15 MBq $ml^{-1}$, and for small spheroids 5.75 MBq $ml^{-1}$, also clearly indicating the greater effect of $^{131}$I-mIBG on larger spheroids.

Spheroids treated with activities of $^{131}$I-sodium iodide up to 6.0 MBq $ml^{-1}$ showed no regrowth delay and none were 'cured'. Similarly, no effect was seen on spheroids treated with non-radiolabelled mIBG up to concentrations of 2 mM, which exceeds by a factor of four the maximum concentrations of $^{131}$I-mIBG used. In addition, the effect of external beam irradiation on spheroids incubated with non-radiolabelled mIBG was indistinguishable from that seen when external beam irradiation alone was used (Figure 5).

It is deduced that the toxicity demonstrated must be due to the incorporation of radioactive mIBG by the neuroblastoma cells, as these control experiments have shown that it cannot be attributed to the $^{131}$I or the mIBG alone nor to an interaction between mIBG and radiation.

The specific regrowth delay produced by external beam irradiation on small and large SK-N-BE(2c) spheroids is shown in Figure 5. As a given dose produces similar effects on spheroids of both sizes, it is deduced that the difference seen with the targeted radiotherapy is due to the physical characteristics of $^{131}$I.

### Discussion

Although the sizes of spheroids chosen in this study, 250 μm and 400 μm, are relatively close together, there is a significant difference in the absorbed fraction, Φ, of $^{131}$I radiation – about 20% for the smaller and 30% for the larger spheroids (Figure 1), i.e. a 50% difference in dose absorption is predicted. While a greater difference pertains if tumours of the order of a millimetre diameter are studied, this is not feasible with the tumour spheroid model. Four hundred μm is in practice at the upper limit of starting size for this system, as SK-N-BE(2c) spheroids tend to disintegrate as their diameter approaches 1 mm.

If, for spheroids of a particular size, the absorbed fraction, Φ, shown in Figure 1 is multiplied by the isoeffective $^{131}$I-mIBG concentration ($TCD_{50}$) shown in Figure 4 a number is obtained which should be proportional to the radiation dose absorbed. If our predictions are correct, then isoeffective $^{131}$I-mIBG concentrations will result in similar absorbed radiation doses in spheroids of different sizes. At 1.15 and 1.25,

**Figure 5** Specific regrowth delay achieved by external beam irradiation of small spheroids (open squares) and large spheroids (filled squares). Symbols are spaced out for clarity, each point represents the result of one experiment. Triangles indicate the radiation doses at which it was not possible to determine the specific regrowth delay as too many spheroids were 'cured' (open triangles, small spheroids; closed triangles, large spheroids). Circles indicate the specific regrowth delay of spheroids achieved by irradiation following incubation with non-radiolabelled mIBG.

the product of Φ and $TCD_{50}$ for small and large spheroids respectively is similar. Thus the results obtained with our *in vitro* model are close to what was predicted by mathematical studies.

The prediction that smaller neuroblastoma micrometastases are more difficult to cure with $^{131}$I-mIBG has serious implications for clinical practice. If targeted therapy cannot be relied upon to eradicate subclinical tumour deposits, $^{131}$I-mIBG must be used as part of a strategy incorporating some other way of sterilising microscopic disease.

Widespread dissemination is a feature of neuroblastoma, and part of the rationale for using biologically targeted therapy. Bone marrow examination in such cases often reveals micrometastases, sometimes just small clumps of cells rather than larger aggregates. Our results predict that it is these deposits which may lead to treatment failure, if undue reliance is placed on $^{131}$I-mIBG therapy. There are several different ways in which this problem may be overcome, including targeted therapy with alternative radionuclides, and combination of $^{131}$I-mIBG with external beam radiotherapy.

Electronic equilibrium is achieved in smaller tumours than is the case with $^{131}$I if radionuclides with emissions of a shorter length are used. One such radionuclide which may prove suitable is the longest lived isotope of the halogen astatine, $^{211}$At. This emits alpha particles with a track length of about 60 μm, adequate to traverse several cell diameters and so kill untargeted cells by cross fire, but short enough to be effective in small diameter micrometastases. In addition, the densely ionising, high linear energy transfer quality of alpha particle radiation means that a lower dose is required to achieve the same effect as $^{131}$I, and gives the added advantage of overcoming the resistance of hypoxic cells. If astatinated benzylguanidine can be produced, its optimal use might be in combination with $^{131}$I-mIBG to achieve maximal levels of tumour cell kill for a range of sizes of micrometastases.

An alternative approach, which is more practical at the present time, is to combine $^{131}$I-mIBG with external beam total body irradiation (TBI). The TBI may eradicate smaller tumour deposits in which there are fewer clonogenic cells, although the dose limits imposed by normal tissue tolerance may prevent cure of larger deposits. If TBI is combined with $^{131}$I-mIBG therapy in the treatment of neuroblastoma, each modality may offset the disadvantages of the other (Wheldon *et al.*, 1988, 1991). An added benefit of this combined strategy is that it should encompass all tumour deposits and so reduce the importance of the problem of heterogeneity of uptake in tumour which may occur with targeted radiopharmaceuticals. This phenomenon, which has not been considered in the present analysis, will be of increasing importance with larger tumours, and may be a limiting factor in the targeted therapy of macroscopic tumours. However experimental studies have shown that, in spheroids at least, uniform distribution of mIBG does occur in contrast to the marked heterogeneity seen with monoclonal antibodies (Mairs *et al.*, 1991*a*).

The optimal scheduling of targeted radiotherapy with TBI has been investigated by O'Donoghue (1991). A set of combined treatment schedules, chosen to be biologically equivalent to a TBI course of seven 2 Gy fractions are evaluated. The tumour effects of these schedules depend on the specificity of targeting, represented by the ratio of the initial dose rate for the tumour cells to that in the dose limiting normal organ, and the heterogeneity of targeting represented by the proportion of tumour cells that escape irradiation by targeted radiotherapy. The optimal schedule predicted varies depending on these factors and the radiobiological parameters of the tumour, but almost always requires a significant component of TBI for maximum therapeutic effectiveness.

In practice a combination regimen consisting of five 2 Gy fractions of TBI and $^{131}$I-mIBG activity calculated for each patient to give a 4 Gy whole body dose (making a total of 14 Gy whole body dose) is predicted to give a high probability of sterilising subclinical neuroblastoma tumours in all size classes. A clinical study to evaluate this strategy in selected patients has recently commenced.

In conclusion, this study produces data indicating that if targeted radiotherapy with $^{131}$I-mIBG is to be used successfully in patients with neuroblastoma, a means of overcoming the relative resistance of micrometastatic disease needs to be incorporated into the therapeutic strategy.

This work was supported by generous grants from The Neuroblastoma Society and The Cancer Research Campaign.

### References

BIEDLER, J.L., ROFFLER-TARLOV, S., SCHACHNER, M. & FREEDMAN, L.S. (1978). Multiple neurotransmitter synthesis by human neuroblastoma cell lines and clones. *Cancer Res.*, **38**, 3751–3757.

CAMPBELL, M.J. & GARDNER, M.J. (1989). Calculating confidence intervals for some non-parametric analyses. In *Statistics with Confidence: Confidence Intervals and Statistical Guidelines*, Gardner, M.J. & Altman, D.G. (eds) pp. 71–79. British Medical Journal: London.

CAMPBELL, M.J. & MACHIN, D. (1990). *Medical Statistics: a Commonsense Approach*. John Wiley and Sons: Chichester.

CARACHI, R., RAZA, T., ROBERTSON, D., WHELDON, T.E., WILSON, L., LIVINGSTONE, A., VAN HEYNINGEN, V., SPOWART, G., MIDDLETON, P., GOSDEN, J.R., KEMSHEAD, J.T. & CLAYTON, J.P. (1987). Biological properties of a tumour cell line (NB1-G) derived from human neuroblastoma. *Br. J. Cancer*, **55**, 407–411.

HOEFNAGEL, C.A., VOÛTE, P.A., DE KRAKER, J. & MARCUSE, H.R. (1987). Radionuclide diagnosis and therapy of neural crest tumours using iodine-131 metaiodobenzylguanidine. *J. Nucl. Med.*, **28**, 308–314.

HUMM, J.L. (1986). Dosimetric aspects of radiolabelled antibodies for tumor therapy. *J. Nucl. Med.*, **27**, 1490–1497.

LASHFORD, L.S., HANCOCK, J.P. & KEMSHEAD, J.T. (1991). Meta-iodobenzylguanidine (mIBG) uptake and storage in the human neuroblastoma cell line SK-N-BE(2C). *Int. J. Cancer*, **47**, 105–109.

LEWIS, I.J., LASHFORD, L.S., FIELDING, S., FLOWER, M.A., ACKERY, D. & KEMSHEAD, J. (1991). A phase I/II study of $^{131}$I mIBG in chemoresistant neuroblastoma. In *Advances in Neuroblastoma Research 3*, Evans, A.E., D'Angio, G.J., Knudson, A.G. & Seeger, R.C. (eds) pp. 463–469. Wiley-Liss: New York.

MAIRS, R.J., ANGERSON, W., GAZE, M.N., MURRAY, T., BABICH, J.W., REID, R. & MCSHARRY, C. (1991*a*). Differential penetration of alternative targeting agents into human neuroblastoma spheroids. *Br. J. Cancer*, **63**, 404–409.

MAIRS, R.J., GAZE, M.N., & BARRETT, A. (1991*b*). The uptake and retention of meta-iodobenzylguanidine by the neuroblastoma cell line NB1-G. *Br. J. Cancer*, **64**, 293–295.

MONTALDO, P.G., LANCIOTTI, M., CASALARO, A., CORNAGLIA-FERRARIS, P. & PONZONI. M. (1991). Accumulation of m-iodobenzylguanidine by neuroblastoma cells results from independent uptake and storage mechanisms. *Cancer Res.*, **51**, 4342–4346.

MOORE, J.V., WEST, C.M.L. & HENDRY, J.H. (1987). Deriving cell survival curves from the overall responses of irradiated tumours: analysis of published data for tumour spheroids. *Br. J. Cancer*, **56**, 309–314.

MOYES, J.S.E., BABICH, J.W., CARTER, R., MELLER, S.T., AGRAWAL, M. & MCELWAIN, T.J. (1989). Quantitative study of radioiodinated metaiodobenzyl guanidine uptake in children with neuroblastoma: correlation with tumour histopathology. *J. Nucl. Med.*, **30**, 474–480.

O'DONOGHUE, J.A. (1991). Optimal scheduling of biologically targeted radiotherapy and total body irradiation with bone marrow rescue for the treatment of systemic malignant disease. *Int. J. Radiat. Oncol. Biol. Phys.*, **21**, 1587–1594.

O'DONOGHUE, J.A., WHELDON, T.E., BABICH, J.W., MOYES, J.S.E., BARRETT, A. & MELLER, S.T. (1991). Implications of the uptake of $^{131}$I-radiolabelled meta-iodobenzylguanidine (mIBG) for the targeted radiotherapy of neuroblastoma. *Br. J. Radiol.*, **64**, 428–434.

POURREAU-SCHNEIDER, N. & MALAISE, E.P. (1981). Relationship between surviving fractions using the colony method, the LD50, and the growth delay after irradiation of human melanoma cells grown as multicellular spheroids. *Radiat. Res.*, **85**, 321–332.

SCHWACHÖFER, J.H.M., CROOIJMANS, R.P.M.A., VAN GASTEREN, J.J.M., HOOGENHOUT, J., JERUSALEM, C.R., KAL, H.B. & THEEUWES, A.G.M. (1989). Radiosensitivity of different human tumour cell lines grown as multicellular spheroids determined from growth curves and survival data. *Int. J. Radiat. Oncol. Biol. Phys.*, **17**, 1015–1020.

TWENTYMAN, P.R. (1982). Growth delay in small EMT 6 spheroids induced by cytotoxic drugs and misonidazole pretreatment under hypoxic conditions. *Br. J. Cancer*, **45**, 565–570.

VOÛTE, P.A., HOEFNAGEL, C.A., DE KRAKER, J., VALDES OLMOS, R., BAKKER, D.J. & VAN DE KLEIJ, A.J. (1991). Results of treatment with $^{131}$I-metaiodobenzylguanidine in patients with neuroblastoma. Future prospects of zetotherapy. In *Advances in Neuroblastoma Research 3*, Evans, A.E., D'Angio, G.J., Knudson, A.G. & Seeger, R.C. (eds) pp. 439–445. Wiley-Liss: New York.

WALKER, K.A., MURRAY, T., HILDITCH, T.E., WHELDON, T.E., GREGOR, A. & HANN, I.M. (1988). A tumour spheroid model for antibody-targeted therapy of micrometastases. *Br. J. Cancer*, **58**, 13–16.

WEST, C.M.L., SANDHU, R.R. & STRATFORD, I.J. (1984). The radiation response of V79 and human tumour multicellular spheroids – cell survival and growth delay studies. *Br. J. Cancer*, **50**, 143–151.

WHELDON, T.E. & BRUNTON, G.F. (1982). Relation of growth delay to cure for experimental tumour systems conforming to poisson cure statistics. *Br. J. Cancer*, **45**, 1–9.

WHELDON, T.E., LIVINGSTONE, A., WILSON, L., O'DONOGHUE, J. & GREGOR, A. (1985). The radiosensitivity of human neuroblastoma cells estimated from regrowth curves of multicellular tumour spheroids. *Br. J. Radiol.*, **58**, 661–664.

WHELDON, T.E., O'DONOGHUE, J.A., HILDITCH, T.E. & BARRETT, A. (1988). Strategies for systemic radiotherapy of micrometastases using antibody targeted $^{131}$I. *Radiother. Oncol.*, **11**, 133–142.

WHELDON, T.E., O'DONOGHUE, J.A., BARRETT, A. & MICHALOWSKI, A.S. (1991). The curability of tumours of differing size by targeted radiotherapy using $^{131}$I or $^{90}$Y. *Radiother. Oncol.*, **21**, 91–99.

WIELAND, D.M., WU, J., BROWN, L.E., MANGNER, T.J., SWANSON, D.P. & BEIERWALTES, W.H. (1980). Radiolabelled adrenergic neuron-blocking agents: adrenomedullary imaging with [$^{131}$I]iodobenzyl guanidine. *J. Nucl. Med.*, **21**, 349–353.